# Expandable Graphite for Flame Retardant PA6 Applications

**DOI:** 10.3390/polym13162733

**Published:** 2021-08-15

**Authors:** Florian Tomiak, Klaus Rathberger, Angelina Schöffel, Dietmar Drummer

**Affiliations:** 1 Institute of Polymer Technology, Friedrich-Alexander-University Erlangen-Nuremberg, Am Weichselgarten 9, 91058 Erlangen, Germany; dietmar.drummer@fau.de; 2 Bavarian Polymer Institute, Friedrich-Alexander-University Erlangen-Nuremberg, Dr. Mack Strasse 77, 90762 Fuerth, Germany; 3 Georg H. Luh GmbH, Schoene Aussicht 39, 65396 Walluf, Germany; klaus.rathberger@luh.de (K.R.); angelina.schoeffel@luh.de (A.S.)

**Keywords:** flame retardant, expandable graphite, PA6, electrical conductivity, thermal conductivity, rheological properties, sample conditioning

## Abstract

A new expandable graphite (EG) type was studied as a flame retardant additive in Polyamide 6 (PA6). The fire behavior was characterized by a cone calorimeter using external heat fluxes of 35, 50 and 65 kW/m^2^, limiting the oxygen index (LOI) and UL-94 burning tests. Additionally, electric and thermal conductivity as well as rheological properties were characterized to provide a general property overview. Fire tests were conducted using dry and humid conditioned samples. Cone Calorimeter tests showed a minimum filling degree of 15 wt.% (8.6 vol.%) EG was required to achieve a significant fire inhibiting effect in PA6 independent of the sample condition. UL-94 fire tests show a V0 classification at filling degrees greater than 20 wt.% (humid) and 25 wt.% (dry), although the associated LOI values of 39% and 38% demonstrate good flammability inhibition. Correlation analyses were conducted to identify major influences given by the sample condition for most important key figures measured in cone calorimeter tests. Accordingly, humid-conditioned samples containing between 2.5 (PA6 + 25 wt.% EG) and 4.2 wt.% (PA6) water were found to reduce the total heat evolved (THE) on average by 16% and the total smoke production (TSP) on average by 22%.

## 1. Introduction

Today, the majority of polymers used in technical applications naturally exhibit poor flame retardancy properties and thus require either molecular or additive modification to lower the probability of a fire hazard. Since intrinsically flame retardant polymers available today mostly exceed the cost of conventional polymers many times over, modification of conventional grades with flame-retardant additives is the most widely used alternative in order to fulfill stringent flame retardancy standards [1,2].

Expandable graphites (EG) are successfully used as flame retardant additives predominantly in polyurethane (PU) foams, gaskets and flame retardant coatings [3]. Compared to other flame retardant additives, expandable graphites are low cost and provide a good flame retardancy effect characterized by low smoke generation and an anti-dripping effect [4]. The flame retardant effect is preliminarily based on the physical formation of a highly expanded, thermally stable residue and can thus be incorporated into different polymeric systems. This effectively slows or stops fire spread by impeding thermal heat feedback from the flame to the substrate, migration of oxygen to the surface, and mass transfer of low molecular weight decomposition products to the gas phase. [5] Despite proven efficiency as a flame retardant in various thermoplastic polymer systems (e.g., PP [6,7,8,9,10,11,12], PE [13,14,15,16], ABS [17], EVA [18,19], PET [20,21], PS [22], PVC [23]), the industrial use of expandable graphite is limited to niche applications. One of the reasons is the low temperature stability of commercially available expandable graphite grades, with maximum processing temperatures typically lower than 230 °C; only some literature is available focusing on this topic [20]. Expandable graphites are produced by intercalating a blowing agent into the graphene layers. Today different blowing agents are used for this process, though commercially used grades are predominantly sulfide blowing agents. [4,24,25]

Expandable graphites also exhibit good thermal conductivity properties. When incorporated into polymeric molding compounds, expandable graphites act in a multifunctional fashion by providing flame-retardant and thermal conductivity improvements simultaneously. This can be particularly advantageous for applications with the need to prevent heat build-up, while also exceeding certain flame resistance classifications like electronic housings, lighting equipment or microelectronic equipment [26]. The thermal conductivity of graphite has excessively been studied in many papers [24,26] and will thus not be discussed in detail.

In this paper a higher temperature-stable expandable graphite grade for use in engineering thermoplastics was fundamentally characterized as flame retardant in PA6. A standard grade PA6 was therefore systematically incorporated by 5, 10, 15, 20 and 25 wt.% expandable graphite. Methods presented include thermogravimetric analysis (TGA) coupled with a Fourier transform infrared spectroscopy (FTIR) gas cell, melt volume flow rate (MVR), thermal diffusivity measurements (hot-disc), electrical conductivity measurements (4-pole method), cone calorimeter tests, limiting oxygen index (LOI) and UL-94 fire testing. Fire testing samples were prepared as dry and humid-conditioned and comparatively evaluated.

## 2. Materials and Methods

### 2.1. Materials, Preparation and Testing

A standard type PA6 was used as a matrix and systematically modified by a high temperature stable expandable graphite type using filling degrees of 5, 10, 15, 20, 25 wt.%.

The PA6 grade B27E (density 1.13 g/cm^3^; melting temperature 220 °C) was provided by BASF AG (Ludwigshafen, Germany), the expandable graphite GHL PX 95 HT 270 (pH value 5–9; exp. rate 200 mL/g; 70% > 50 mesh ASTM (=300 μm)) was provided by LUH GmbH (Walluf, Germany). PA6/ EG formulations were prepared using a twin-screw extrusion machine DSE ZSE HP 27 from Leistritz GmbH (Nuremberg, Germany). Temperatures were controlled between a die and polymer feeder at 230 °C to 220 °C. The screw configuration used had three shear and kneading zones. A screw speed of 100 r/min and a material throughput of 8 kg/h was used. The strand was drawn off through a water bath, granulated by cold drawdown and dried afterwards. All compounds were injection molded (mold: 115 × 115 × 4 mm^3^) using an Allrounder 370 V injection molding machine from Arburg GmbH & CoKG (Loßburg, Germany). Aggregate temperatures were controlled between 230 °C (die) to 220 °C; injection speed at 60 mm/s; the mold temperature was 80 °C. Processing parameters were determined experimentally. All samples needed were prepared from the injection molded plates. Samples were conditioned dry (70 °C; vacuum) and humid (70 °C; 62% rel. humidity until the weight was constant). The total water load for dried and humid conditioned samples PA6 and PA6 + 25 wt.% was 0.4 ± 0.0/0.4 ± 0.0 and 3 ± 0.1/2.5 ± 0.1 wt.%, respectively.

#### 2.1.1. Microscopy

To identify the overall particle distribution within the injection molded plate, samples were taken at distinct in-plate locations. Samples were grinded and images were taken using reflected light microscopy. Additionally, scanning electron microscope (SEM) studies were conducted using the device SEM Ultra Plus from Zeiss (Oberkochen, Germany). The samples were spattered with a platinum-palladium mixture.

#### 2.1.2. Thermal Analysis

TGA/ DSC samples (10 mg ± 1 mg) were heated from 50 °C to 800 °C at a constant heating rate of 20 °C/min using a STA Jupiter F3 from Netzsch (Selb, Germany). The atmosphere was nitrogen at a flow rate of 70 mL/min. The transfer line between TGA/ DSC and FTIR was temperature-controlled at 230 °C, the FTIR gas chamber at 200 °C. A FTIR gas unit Tensor 2 from Bruker (Billerica, MA, USA) was used.

#### 2.1.3. Fire Testing

Cone Calorimeter (ISO 5660-1), UL-94 (DIN EN 60695-11-10/20) and Limiting Oxygen Index (LOI) (DIN EN ISO 4589-2) fire tests were used to characterize the burning behavior. All devices were from Netzsch Taurus GmbH (Weimar, Germany) with no specific device code. Cone Calorimeter tests are probably the most important method for researching and characterizing fire behavior. Analytics monitor the heat development of fully evolved fires over time, generating comprehensive insights into flame retarding mechanisms and provide the basis for fundamental evaluations through key performance figures. The heat release rate (HRR), total heat release rate (THR), mass loss rate (MLR), time to ignition (T_ign_) and smoke production rate (SPR) are the most important key figures to be named. Furthermore, average or ratio values are also frequently used to gain deeper insights, such as the average rate of heat emission (AHRE) or the Maximum AHRE (MAHRE), fire propagation index (FPI) or fire growth rate (FIGRA). The FPI, defined as T_ign_/pHRR and the FIGRA, defined as the maximum of HRR(t)/t are used to compress complex burning characteristics gained in cone calorimeter tests and provide a practical basis for comparison reasons only. [27,28,29] For cone calorimeter tests conducted within this study 100 × 100 × 4 mm^3^ samples were prepared and tested at external heat fluxes of 35, 50 and 65 kW/m^2^. All tests were repeated at least three times. Averaged curves are presented. 

Limiting Oxygen Index (LOI) tests are commonly used to characterize flammability properties of polymers, whereas the oxygen index (OI) represents the lowest atmospheric oxygen content needed to support an ongoing, visible flame. A 50 W propane flame is applied six times for 5 s each in a candle-like setup. The LOI value is predominantly used as a comparative value. Sample geometries used were 125 × 10 × 4 mm^3^. 

UL-94 tests are used to conduct self-distinguishing properties. The UL-94 V test exposes a 50 W flame to a vertically clamped sample for two times 10 s. Depending on the fire characteristics observed, four classifications are then to be assigned based on the standard specifications: V-0, V-1, V-2 and HB. V-0 represents the highest classification characterizing the fire response as non-burn-dripping and almost instant self-extinguishing behavior. V-1 is a transitional classification and allows longer afterburn times, but no burn dripping. V-2 classifications show low self-extinguishing behavior, mostly considered as not sufficient. Self-extinguishing properties are at a minimum and permit not only long afterburning times but also burn dripping. Sample geometries used for these tests were 125 × 13 × 4 mm^3^. All tests were conducted in accordance to test procedure standards.

#### 2.1.4. Electrical, Heat and Thermal Conductivity, Heat Capacity and Density

Electrical measurements were conducted using a ring electrode device Milli-TO3 from Fischer Elektronik GmbH (Luedenscheid, Germany) with an inner diameter of 10 mm and a measuring gap of 1mm. The measuring range of the device is 10 to 10 Ohm at 100 V measuring voltage. Two different sample variants were used for electrical conductivity measurements. For both samples, an injection-molded plate geometry with 50 mm × 50 mm and an initial thickness of 4 mm was used. Measurements were performed on freshly molded samples with the original plate thickness of 4 mm. Additionally, in order to exclude edge layer effects typically evident in injection-molded parts, we removed the edge layer by 0.7 mm on both sides. The total sample thickness was 2.6 mm. Tests were conducted in accordance with standards. Heat conductivity measurements were performed using a Nanoflash device LFA 447 from Netzsch GmbH (Selb, Germany) at room temperature. Sample geometries were 12.7 × 12.7 × 4 mm^3^. For density measurements a gaspyknometer device AccuPyc 1330 from Micromertics Instrument Corp. (Norcross, GA, USA), for heat capacity measurements Calvet Calorimeter C80 from Setaram Instrumentation Inc. (Caluire, France) was used. All samples were dried before testing.

#### 2.1.5. Viscosity Measurements

Viscosity measurements were conducted by rotational viscositmeter and MVR measurements. Therefore, a rotational viscositmeter device Discovery HR-2 from TA Instruments Inc. (New Castle, DE, USA) was used. Tests were performed at 230 °C and nitrogen atmosphere. To destroy possible agglomerates a pre-soak-time of 2 min and a pre-share duration of 1min at a frequency of 1 Hz and 1% strain was applied before to every measurement. The test setup was a plate-plate experiment with a diameter of 20 mm. Afterwards a second soak-time of 2 min was applied. Measurements were conducted at 1% strain, a frequency of 0.01-100 Hz and measurement gap of 1 mm. MVR measurements (DIN EN ISO 1133) were conducted twice at 230 °C and 2.16 kg (conditioning dry; 70 °C at vacuum) using a MVR-device type 4106 from Zwick (Ulm, Germany).

## 3. Results

### 3.1. Microscopy

**Figure 1:**
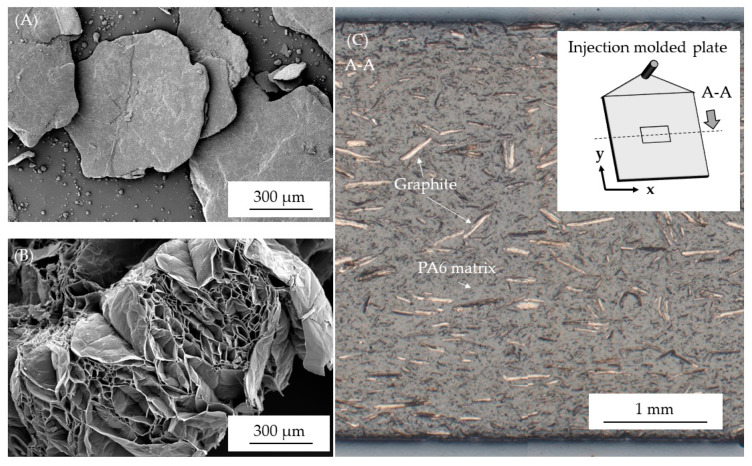
SEM image of expandable graphite (**A**) before expansion and (**B**) after expansion; (**C**) light microscopy image of particle distribution for PA6 containing 20 wt.% EG.

Figure 1 shows SEM and light microscopy images of net EG as well as incorporated in a PA6 matrix. Expandable graphites are plate-like particles (Figure 1A) and expand perpendicular to the plane when a critical temperature is reached (Figure 1B). After expansion the structure is foam-like, but rather brittle with low mechanical stability. EG was incorporated into PA6, injection molded and tested afterward. Light images were taken from multiple locations, whereas one representative image is represented in Figure 1C. Please note, the given image is representative in matters of distribution and particle orientation for all filling degrees conducted. 

The images revealed a homogenous particle distribution throughout the injection molded plate with no visible segregations or voids. Particle orientations were predominantly in meltflow-direction, with no visually detectable differences between the sampling positions.

### 3.2. Thermal Decomposition and Gas Analysis

TGA/DSC-FTIR measurements were conducted for all formulations tested and repeated three times. For reasons of clarity only TGA curves for net EG, net PA6 and PA6 containing 20 wt.% EG were plotted. Major key figures for all formulations, including standard deviations, can be found in Table 1. The decomposition process of PA6 has been comprehensively analyzed by many authors and will therefore not be discussed in detail within this paper. An excellent overview can be found in [30,31,32,33]. 

**Figure 2:**
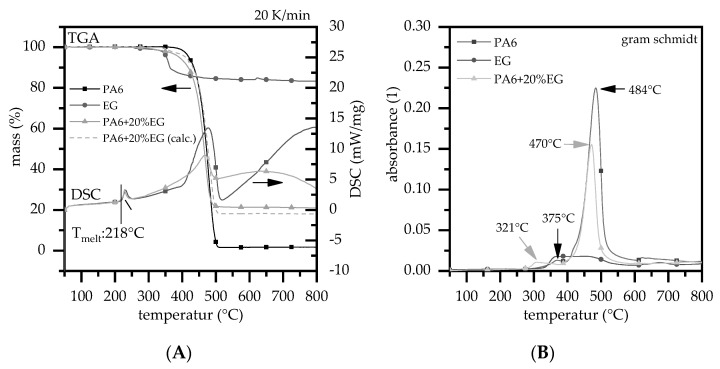
Measurements of PA6, expandable graphite (EG) and PA6 + 20 wt.% EG. Heating rate 20 K/min; nitrogen atmosphere; (**A**) TGA, DSC; (**B**) gram schmidt.

DSC measurements show a melting temperature at 218°C for all formulations tested, whereas the melting enthalpy decreased with increasing filling degree within the expaction range. Accordingly, for PA6 and PA6 containing 20 wt.% EG a melting enthalpy of 76 J/g and 61 J/g was measured (Figure 2). This fits well to a calculated value for PA6 containing 20 wt.% EG of 60.8 J/g (76 J/g × 0.8). 

**Figure 3:**
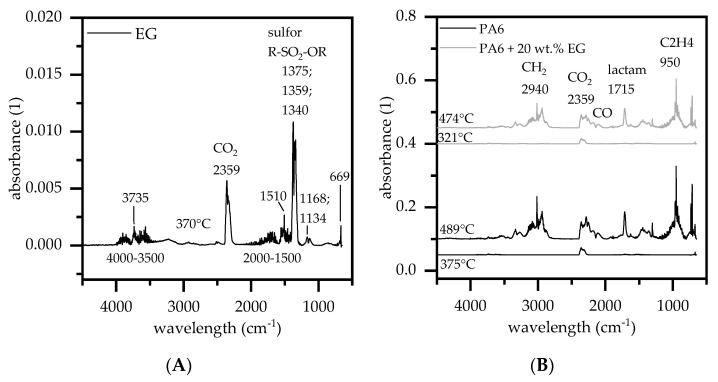
IR gasphase spectra taken from TGA-FTIR coupled measurements at 20 K/min and nitrogen atmosphere; (**A**) FTIR spectra of expandable graphite (EG) at 370°C; (**B**) FTIR spectra of PA6 and PA6 + 20 wt.% EG.

As expected, net PA6 measured decomposed within one major gravimetrical step, revealing an onset temperature at 393 °C (T99%) and a peak decomposition temperature at 477 °C (gram schmidt peak at 484 °C; the delay corresponds to the physical gas transfer between TGA and FTIR). The residue of net PA6 at 800 °C was less than 1%. Major gas products for PA6 decomposition known from the literature could also be detected: lactam (1715 cm^−1^), NH_3_ groups (950, 3330 cm^−1^), CH_2_ (2940, 1440, 730 cm^−1^), CH_4_ (3016 cm^−1^) ammonia groups (930, 965 cm^−1^) as well as CO_2_ (2360 cm^−1^, 670 cm^−1^) and water (3000–4000 cm^−2^) (Figure 3B). [31] An additional, non-gravimetrical peak was found for net PA6 through FTIR measurements at 375 °C and for PA6 +20 wt.% EG at 321 °C showing CO_2_ and water release. This might indicate a partial breakdown of caprolactam due to hydrolysis to form carboxylic acid, followed by a further breakdown to give CO_2_ and H_2_O [33]. Since the samples were pre-dried and no gravimetric mass loss step occurred in TGA curves, we attribute this effect to minimal residual water in the polymer.

Net EG shows two gravimetrical decomposition steps with a minor step at DTG_peak_ 253 °C (−0.75%) and a major step DTG_peak_ 349 °C (−15%) as well as a total residue at 800 °C of 83%. The minor step is mostly CO_2_ and water, whereas also some traces of an epoxy resin (3735 cm^−1^) were found. Due to a plateau formation between 253 °C (−0.75%) and 307°C (−1%) an onset temperature defined by 1% mass loss could not be clearly determined. Isothermal tests revealed no expansion until 290 °C and only minor expansion until 310 °C in nitrogen atmosphere. For production purposes, the first step is therefore considered of minor importance. For the second decomposition step gas analytics reveal CO_2_ and sulfur bonds (1340, 1359, 1375 cm^−1^) as major decomposition products, indicating a sulfur based blowing agent. This is little surprising, thus sulfur based blowing agents are a typical component for expandable graphites [3,34,35].

Furthermore, as already discovered at lower temperatures, traces of epoxy resins were found. Expandable graphite reduced the decomposition onset temperature for PA6/EG formulations due to earlier EG blowing reactions. With higher filling levels, the incorporated particles increasingly dominate the overall onset temperature reaching 316 °C for PA6 + 25wt.% EG. However, the subsequent decomposition characteristic measured does not change substantially, marking a peak in gravimetrical decomposition speed between 471 °C and 477 °C for all formulations tested. FTIR gas analytics reveal similar gas product formations, indicating no major changes in chemical decomposition pathways. SO_2_ is not visible in FTIR images of PA6/EG formulations and is thus considered of minor importance. Accordingly, observed flame retarding effects can be solemnly attributed to physical EG expansion. Higher filling degrees of EG result in an increase residual mass at 800 °C. Measurements fit well to calculated residues conducted from net ingredients. Calculated and measured key figures can be found in Table 1. 

### 3.3. Fire Testing

**Figure 4:**
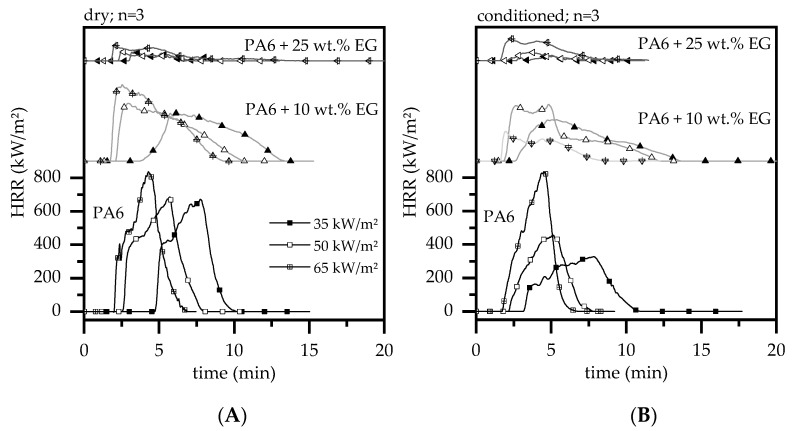
Heat release rate (HRR) over time of selected samples; external heat fluxes conducted were 35, 50, 65 kW/m^2^; (**A**) dried samples (**B**) humid-conditioned samples.

Figure 4 illustrates the Cone Calorimeter results conducted at an external heat flux of 35, 50 and 65 kW/m^2^, for dry and humid conditioned test samples. For reasons of clarity, the plot is limited to a selection of representative formulations. An overview of the main parameters, including standard deviations, can be found in Table 2

Once ignited, net PA6 burns with a quick acceleration in HRR, a high pHRR (Figure 4) and a high average burning rate. By gradual addition of EG in PA6/EG formulations the burning rate decreases significantly. Particularly high filler levels of expandable graphite promote the formation of a voluminous, thermally stable residue, efficiently acting as barrier between polymer and heat source and thus lowering the fuel supply. Cone calorimeter tests are particularly sensitive to strong barrier formation, leading to a characteristic decrease in heat development measured and differences in the pHRR. Measurements conducted for PA6, PA6 + 10 wt.% EG and PA6 + 25 wt.% EG revealed a decrease in pHRR of 899/706/716 kW/m^2^ for dry and 871/ 564/ 371 kW/m² for humid conditioned samples when a heat flux of 50 kW/m^2^ was applied. Other heat fluxes were applied accordingly; key figures are given in Table 2. Particularly strong pHRR reduction can be identified for humid-conditioned samples. Water incorporation in humid-conditioned and dried samples was measured by carl fischer titration to be 0.4 wt.% and 3 wt.% -2.5 wt.% for PA6 and PA6 + 25 wt.% EG. The total weight of polymer for dry and humid-conditioned samples was identical. The high heat capacity of water as well as the formation of water vapor during decomposition acts as heat sink and pyrolysis gas diluter and thus lowering the average burning rate. Specifically lower heater fluxes of 35 and 50 kW/m² seem to benefit from this effect, whereas for 65 kW/m^2^ dry and humid conditioned samples achieved nearly identical pHRR values.

When lower external heat fluxes were applied T_ign_ delayed significantly, whereas dried samples seemed to ignite even later (Figure 4). The relatively early ignition of humid conditioned samples is caused by bubbling, which promotes pyrolysis gas transfer and quickens heat absorption due to higher surface areas. Dry PA6 on the other hand formed a charred skin, which was observed to efficiently retain pyrolysis gases in early decomposition stages. Please note, major characteristic changes of the burning behavior at other loading levels did not appear. Graphs shown in Figure 4 can thus be interpreted as representative selection all Cone calorimeter tests conducted.

**Figure 5:**
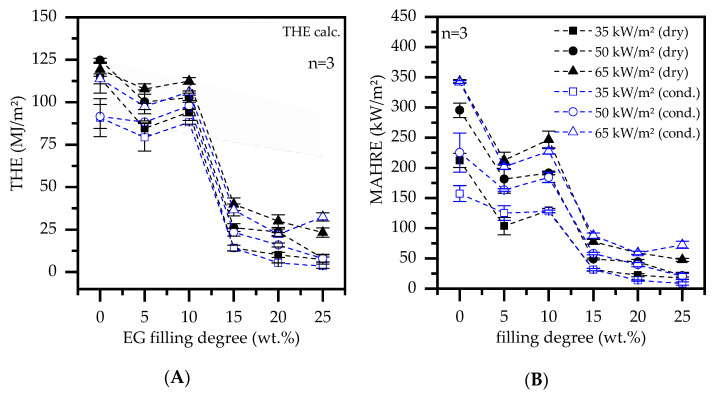
(**A**) comparative plot of the EG filling degree versus the total heat emerged (THE) for all samples tested. (**B**) maximum of the average heat release rate (MAHRE) versus filling degree.

Tests simulate enforced flaming conditions, which eventually lead to complete combustion for most samples. When strong expansion with a highly effective thermal barrier formation occurs, the THE can sometimes also be found to decrease disproportionally greater than expected from filler substitution. For all PA6/EG formulations tested within this study, we found a disproportional decrease in THE for filling degrees greater or equal to 15 wt.% EG (Figure 5A). The principal effect-characteristic occurred independent of the applied heater flux and the sample conditioning state. Two factors were attributed to the measured drop in THE: (1) When a critical barrier effect is reached, decomposition onset-temperatures are no longer reached in deeper layers, leading to a large proportion of non-pyrolysed polymer. Thus, fewer pyrolysis gases are evaporated fueling the fire (2). The resulting rate of burning combustion was partly too low for O_2_ detection, thus a certain deviation to the true value must be considered. An increasing thermal barrier also reduces the average rate of heat release (AHRE), which is typically compared by the maximum of the average rate of heat release (MAHRE). Excluding the formulation PA6 + 10 wt.% a regressing characteristic can be observed (Figure 5B). Since filling degrees greater 15 wt.% only show minor improvements in MAHRE, a maximum in filling degree/ performance ratio is achieved for this formulation.

**Figure 6:**
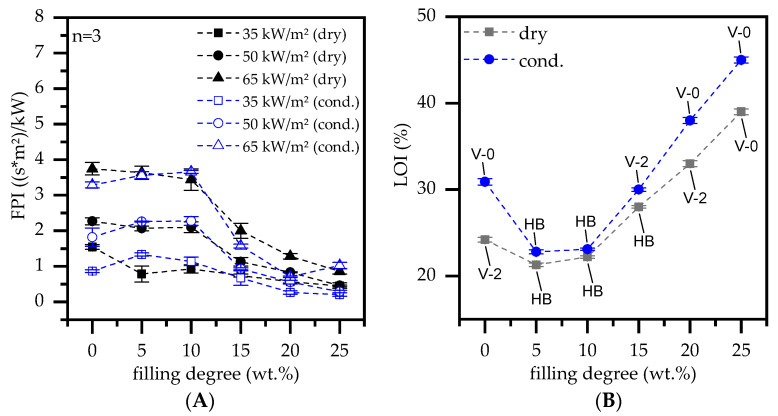
(**A**) fire propagation index (FPI = Tign/pHRR) and (**B**) limiting oxygen index (LOI) versus filling degree of expandable graphite (EG) in a PA6 matrix.

The fire propagation index (FPI) is defined as ratio T_ign_/ pHRR and allows a comparative assessment between complex cone calorimeter results [36]. A low FPI indicates low fire propagation and thus stands for better flame retardant properties measured in cone calorimeter tests. PA6 formulations containing less or equal to 10 wt.% do not change the FPI, but rather form a stationary plateau (Figure 6A). Depending on the external heat flux applied, the plateau appears at a different order of magnitude, which indicates an identical fire response characteristic only dependent on the ignition source. For filling degrees greater or equal to 15 wt.% EG in PA6, the FPI improves significantly. Accordingly, the FPI nearly halves for all external heat fluxes applied, exhibiting a converging characteristic. Higher filling degrees of 20 wt.% and 25 wt.% further reduce the FPI, yet with a lower performance increase. Since this fits well with observed changes in the THE reported earlier (Figure 5A), a filling degree of 15 wt.% is considered as lower limit to achieve a noticeable flame retarding effect. 

LOI and UL—94 burning tests showed a strong sensitivity for dry and humid-conditioned samples (Figure 6B). Bubbling and strong melt dripping (rather melt breaking) occurred while testing for humid conditioned samples, whereas dried samples showed better structural stability and some char-skin formation (Please note the sample thickness of 4 mm). This resulted in humid-conditioned PA6 achieving a relatively high LOI value of 31% as well as a V-0 classification, whereas dry PA6 only achieved an LOI value of 24% and a V-2 classification. Depending on the EG filling degree tested, PA6 formulations showed different behaviors. Low filling degrees ≤ 10 wt.% resulted in rather burn breaking, while a complete burn-down of the sample could be observed. Accordingly, the char residue did not built-up quickly and dense enough in order to provide a sufficient thermal barrier. Please find burning times in Table 2. With increasing filling degrees of ≥15 wt.% the char residue constantly improved in early flame exposure stages leading to a constant reduction in burning times. Though, V-0 classification were only achieved for PA6 containing 20 wt.%/ 25 wt.% EG for humid conditioned and 25 wt.% for dried samples due to the effects described. A better classification for humid conditioned samples at a loading level of 20 wt.% was solemnly due to no cotton ignition. 

Please note, higher exposure times than 20 s given by the standard testing procedure might change classifications achieved. Breaking sample-fragments leave an undefined surface exhibiting only some char residue at the lower end of the sample. Thus, repeated flame exposure encounters a geometrically undefined and only partially, char protected sample. To improve the flame retardancy performance in UL-94 test conditions, synergistic systems might be appropriate to compensate this disadvantage. Further studies are therefore needed.

### 3.4. Correlation Analysis 

**Figure 7:**
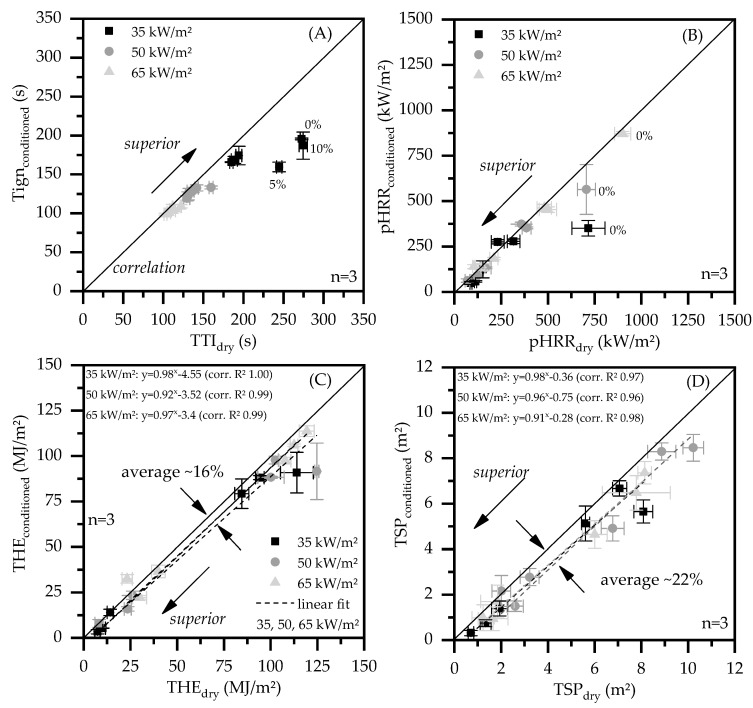
Correlation between all dried and humid conditioned samples tested. (**A**) time to ignition—TTI; (**B**) peak of the heat release rate—pHRR (**C**); total heat emitted—THE (**D**) total smoke production—TSP.

Depending on the condition state of the sample, certain differences in cone calorimeter key figures could be identified (Figrue 7, Table 3). Accordingly, a correlation analysis was conducted for T_ign_, pHRR, THE and TSP including dried and humid conditioned samples. The ignition times (T_ign_) measured for dry and humid samples show good correlations at external heat fluxes of 50 and 65 kW/m^2^, thus T_ign_ is considered independent of the conditioning state (Figure 7A). As expected, higher external heat fluxes lower the T_ign_ measured, whereas the given filling degree within a test setup is considered of minor importance. CC tests conducted at an external heat flux of 35 kW/m^2^ on the other hand showed different characteristics. Specifically dry PA6 formulations containing less or equal to 10 wt.% EG tend to ignite later then humid conditioned samples. As discussed earlier, this effect is attributed to the lack of water evaporation. A skinny, gastight char forms in early PA6 decomposition stages not effected or destroyed by bubbling. At low external heat fluxes, the skin layer shows a more or less good temperature stability and keeps considerable amounts of pyrolysis gases from entering the gas phase. This effect can be observed for net PA6 as well as PA6 containing lower loading levels EG. 

Except for net PA6, the pHRR measured shows good correlations for dry and humid conditioned samples (Figure 7B). As already illustrated in Figure 4, dry PA6 tends to combust more explosively and yields a higher pHRR when external heat fluxes of 35 and 50 kW/m^2^ were applied. Measurements at an external heat flux of 65 kW/m^2^ on the other hand correlate appropriately. We attribute this effect to the cooling effect given by incorporated water, which acts as heat sink and through gas phase delusion. When expandable graphite is added, the filler dominates the decomposition behavior, whereas sample conditions are of less importance for this key figure.

The total heat evolved (THE) (Figure 7C) as well as the total smoke production (TSP) (Figure 7D) show a different behavior. Results for all external heat fluxes applied as well as all formulations used correlate properly within linear fits (R^2^ < 0.96), though appear systematically lower than the equilibrium expectation. An average deviation was determined for both key figures, which can be interpreted as orientation value. Accordingly, humid conditioned formulations achieved an average of ~16% lower THE and ~22% lower smoke production. Obviously, the true values are somewhat dependent on the individual formulation given.

### 3.5. Electrical and Thermal Conductivity

**Figure 8:**
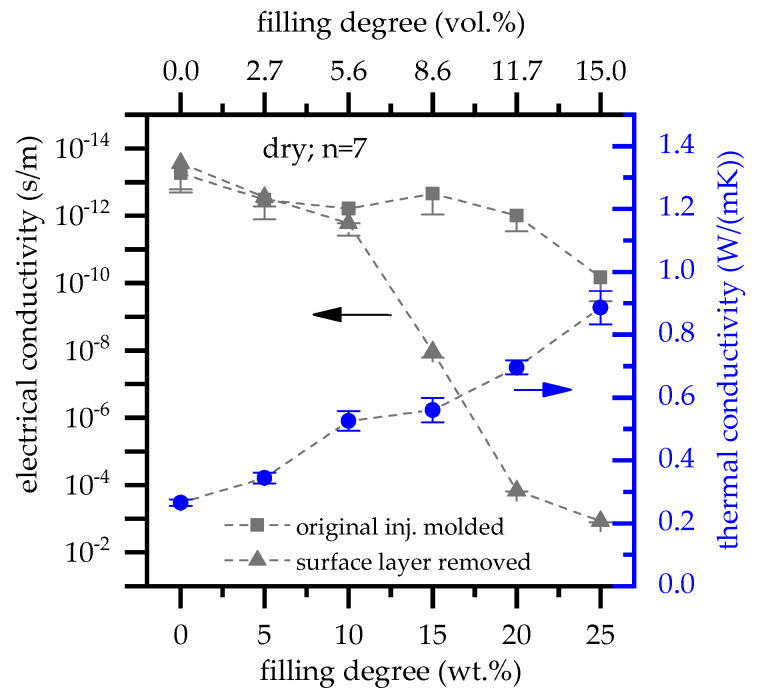
Electrical and thermal conductivity of PA6 filled with expandable graphite.

As expected, the electrical and thermal conductivity increases with increasing filler content (Figure 8). The electrical conductivity measurements were carried out using two sample types: a non-processed sample, referring to the originally injection molded plate with an initial thickness of 4 mm; a proceeded sample, referring to proceeded through surface removal in order to exclude typical surface layer effects found in injection molded parts. Differences found between non- and proceeded samples are considerable. While for non-processed samples no percolation was reached at any filling degree tested, proceeded samples achieved percolation at filling degrees greater 20 wt.% (11.7 vol.%) with ≥1 × 10^−4^ S/m. Since particle orientations found in edge and core layers did not significantly change (Figure 1 and Figure 8), we attribute this effect to the absence of the edge layer. In order to reach the percolation threshold, graphite particles must form a continuous pathway through the sample. Removing surface layers reduces the sample thickness and thus increases a connecting probability. 

Additionally, injection molded parts might sometimes exhibit different surface layer characteristics as a result of processing conditions like mold temperatures, injection speed, pressure, etc. Resulting flow characteristic and solidification behavior generates other crystal structures, thin polymeric surface layers or specific particle orientations. This might change electrical isolation properties, especially for low voltages applied.

Thermal conductivity was calculated from temperature conductivity, density and heat capacity measurements (Figure 8). Expandable graphite-filled polyamides show a nearly linear correlation between thermal conductivity and filler content. Accordingly, the highest values were measured for PA6 containing 25 wt.% (15 vol.%) EG achieving ~0.9 W/(m*K). This values fit well to findings in other studies [26,37,38].

### 3.6. Melt Viskosity

**Figure 9:**
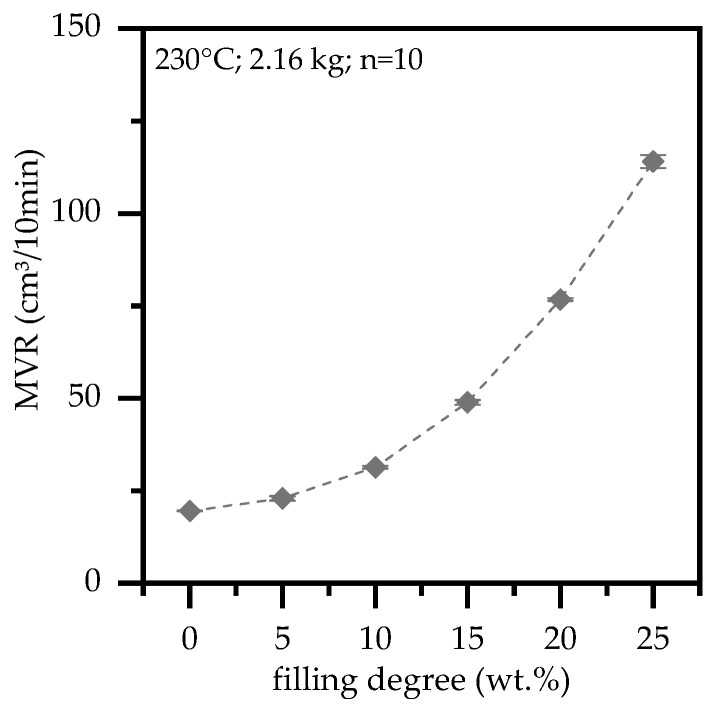
Melt-volume-rate (MVR) for PA6 filled with 0–25 wt.%; temperature 230 °C 2.16 kg.

The melt viscosity properties were characterized by melt-volumen-rate (MVR) and plate-plate rotational viscosimeter measurements (Figure 9). Higher filling degrees of EG in a PA6 matrix exhibited significant lower viscosities, whereas MVR measurements even indicated an exponential character. Accordingly, MVR measured for PA6 containing 25 wt.% was 114 cm^3^/10min, which is nearly six times higher than net PA6 with 20 cm³/10min. This finding fits well to lower injection preasures in the injection molding. We attribute this to a better overall flowability.

**Figure 10:**
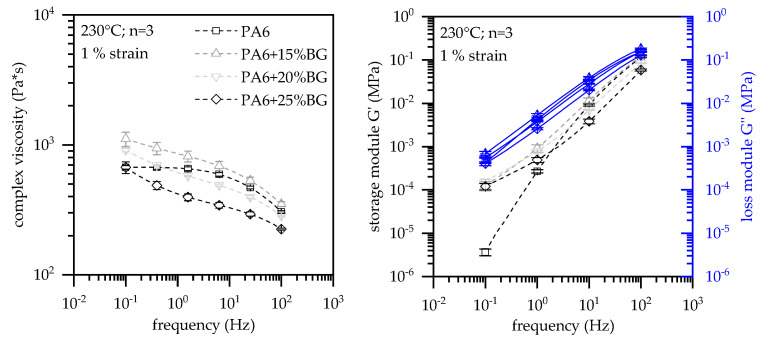
Rotational viscosity measurements; plate-plate setup; 230 °C at 1% strain.

A similar characteristic can be found in rotational viscosimeter measurements (Figure 10). Net PA6 exhibits a Newtonian plateau for low shear rates, whereas for none of the compounds a stationary behavior could be identified within the frequency range meassured. The complex viscosity of EG filled PA6 indicate a strong sheer thinning effect, whereas for higher sheer rates the viscous behavior of PA6 seems to dominate the melt flow characteristic. Curve characteristics initially imply an increase of the melt viscosity for filling levels of 15 wt.% EG, whereas for 20 wt.% and 25 wt.% a further decrease in melt viscosity can be identified. The sheer thinning effect as well as the initial increase in the complex viscosity for PA6 containing 15 wt.% EG versus net PA6 can be attributed to a characteristic property change in the storage module specifically when low frequencies are applied. Higer filling degrees proportionally reduce both storage and loss module and thus lead to a further decrease in the complex viscosity identified. Two effects are attributed to the reduction in viscosity: (1) Due to the absence of surface modification of the given graphite grade, as well as the typically smooth graphite surface (Figure 1A), the given bond between particles and matrix polymer is small and thus promotes a low melt viscosity. (2) Graphite is also known for its lubricating properties and is therefore used as flow promoter. Bonding forces in between the graphite layers are lower than the shear introduced during processing and thus tend to slip when a critical shear rate is reached. This also explains the high MVR values measured, which presumably result in part from wall gliding effects.

## 4. Discussion

The expandable graphite grade showed an isothermal temperature stability of up to 290 °C. No expansion reaction was observed below this temperature, thus providing sufficient temperature stability for processing is assumed. Larger graphite particles were found to be superior in matters of flame retardant performance [39]. Thus, a graphite particle mesh of 70 was selected, which can be generally considered as appropriate.

PA6/EG formulations provided excellent results in cone calorimeter and LOI test, though showed less efficiency in UL-94 tests. As it has been reported for other polymeric systems, the general mode of action was also found to be exclusively physical, resulting in a lower burning rate, lower smoke production and a significant drop of the pHRR and THE (e.g., [14,16,40]). No changes in gas phase decomposition products were found confirming a purely physical expansion mode with no chemical interactions between polymer and filler. Strong expansion of the EG particles provide a voluminous, thermally stable char, specifically advantageous in cone calorimeter tests [5,14]. 

Higher filling degrees improved all relevant key figures measurable in cone calorimeter tests. Particularly in early phases of fire development, the protective residue is not yet fully developed, giving only some protection from an external heat source. Further residue development forming volumes, thermally stable barrier slowly improves the barrier providing an subsequently reduces the HRR. This was characterized in cone calorimeter tests by an early peak heat release rate (pHRR) and a subsequent drop in heat development as response to an improved thermal barrier. Similar characteristics were also reported elsewhere for other polymeric systems (e.g., [14,17,19]). In order to provide good flame inhibiting properties in cone calorimeter tests a minimum filling degree of 15 wt.% was identified. 

Other mechanisms such as bubbling due to the presence of moisture, water evaporation, skin formation in case of dried samples and increased thermal conductivity also occurred. Results showed that ignition times for dry-conditioned samples delayed significantly, when an external heat flux of 35 kW/m^2^ was applied, whereas no differences were detected for 50 and 65 kW/m^2^. The delay was mainly caused by the formation of a carbon skin layer, which efficiently prevented a constant pyrolysis gas flow. Bubble formation, as observed for humid-conditioned samples, as well as high EG filling degrees predominantly destroyed the thin skin layer and thus neglected the inhibition effect. For net PA6, humid-conditioned samples revealed a lower peak heat release rate (pHRR) when external heat fluxes of 50 and 65 kW/m^2^ were applied. Studies have found that dilution effects caused by inert gases only lower flammability limits at high volumetrically proportions [41,42]. Water simultaneously works as heat sink and by delusion, whereby both effects achieve a relatively higher effect when lower pyrolysis gas flows occur. Higher external heat fluxes increase the pyrolysis gas flow and reduce the fire retardant effect due to the presence of water. On the contrary, the total heat generation (THE) and the smoke generation (TSP) showed verifiable lower results for humid-conditioned samples. As an average value, humid-conditioned samples achieved a reduction in THE of −16% and in TSP of −22% compared to dry-conditioned samples.

In flammability tests such as the LOI or self-extinguishing tests such as the UL-94 V burning test, the ignition source and hence the heat flux is proportionally smaller than the sample. As expected, higher EG filling degrees resulted in an increasing LOI value due to an improved residue formation. Additionally, relative to the pyrolysis gas flow, dilution and heat sink effects have a stronger impact. LOI tests for PA6 and PA6/ EG formulations resulted in systematically higher OI values for humid-conditioned samples, revealing a sensitivity in the given test setup. The biggest deltas were found for net PA6 and PA6 containing 25 wt.% EG, giving OI values for dry and humid conditioned samples of 24/31% and 39/45%. The improved thermal conductivity is also likely to have an effect on the ignition process. Unlike cone calorimeter setups, LOI and UL-94 tests are based on a small ignition source applied on one end of the sample. Thermal conductive materials can thus dissipate thermal energy from the ignition area, lowering melt temperatures and hence pyrolysis gas flows. This might also contribute to the sharp increase in measured LOI values. More research is needed in order to separate expansion and thermal conductivity effects.

UL-94 tests for PA6/ EG formulations only achieved high classifications at high filling levels (V-0 for PA6/20, 25 wt.% EG (humid) and PA6/25 wt.% (dried)), whereas the reason for different classifications were solemnly burn dripping/breaking occurring for dried samples. We attribute the necessity of high filling degrees to an insufficient char formation in early stages of the flame exposure. Additionally, we observed melt breaking of sample fragments while and after flame exposure, leaving the remaining specimen unprotected. This is a major disadvantage compared to (well optimized) phosphoric flame retardant additives. Here, when onset temperatures are reached the flame suppressing mechanism acts regardless the exposure time, and thus achieving high UL-94 classifications at lower filling degrees. 

It must be added that the formed residue has little mechanical stability and tends to crumble. In non-horizontal test arrangements, this effect is likely to have a negative impact. Synergistic systems to stabilize the residue might relativize this disadvantage.

## 5. Conclusions

Expandable graphite (EG) was used as flame retardant additive in Polyamide 6 at systematically varied loading levels. Cone Calorimeter, LOI and UL-94 tests as well as rheological, electrical, thermal measurements were conducted and discussed. For fire tests, PA6/EG samples were conditioned both humid and dry, and corresponding results are presented. Major findings are summarized as follows:

- No expansion occurred for temperatures below 290 °C. Process stability is assumed up to this temperature.
- PA6/ EG formulations were found to perform specifically well in cone calorimeter tests. It was found that a minimum filling level of 15 wt.% is required to achieve adequate results.
- In cone calorimeter tests, sample conditions were found to predominantly effect the parameters total heat emitted (THE) and total smoke production (TSP). As an average over all tests conducted, humid-conditioned samples were found to reduce the THE by 16% and the TSP by 22%. Sensitivity of the time to ignition (T_ign_) and the peak heat relelase rate (pHRR) were specifically found for net PA6 as well as when low heat fluxes were applied.
- PA6/ EG formulations were found to work less sufficient in flammability tests. Filling degrees of 20 wt. for humid-conditioned samples and 25 wt.% for dried samples achieved a V-0 UL-94 classification.
- Higher filling degrees were found to increase the heat conductivity of net PA6 versus PA6 containing 25 wt.% EG from 0.27 W/(m*K) to 0.89 W/(m*K).
- The percolation threshold was found to be dependent on the preparation method. Injection molded plates did not exceed electrical conductivity, whereas plates which were processed to remove the surface layers achieved percolation for filling degrees between 10 wt.% (5.6 vol.%) and 20 wt.% (11.7 vol.%).
- Viscosity measurements showed an increase in MVR for higher filling degrees, achieving 114 cm^3^/10 min for PA6 containing 25 wt.%. Rotational viscosity measurements indicate a sheer thinning effect by EG incorporation. For filling degrees of 15 wt.% EG the complex viscosity increases, whereas PA6 containing 20 wt.% and 25 wt.% subsequently decrease the complex viscosity.

## Figures and Tables

**Table 1:** TGA results—summary.

No.	PA6/EG wt.%	t_onset_ (99%) °C	DTG-Peak °C/min	Residue %	Residue Calc. %
1	0/100	307 ± 1	349 ± 3	83	-
2	100/0	393 ± 2	477 ± 2	0.8 ± 0.1	-
3	95/5	373 ± 1	476 ± 2	5.7 ± 0.2	4.9
4	90/10	338 ± 5	475 ± 1	10.1 ± 0.2	9.0
5	85/15	332 ± 0	477 ± 1	12.7 ± 0.1	13.1
6	80/20	333 ± 4	471 ± 2	18.7 ± 1.1	17.2
7	75/25	316 ± 3	471 ± 1	21.7 ± 0.5	21.4

**Table 2:** UL-94 testing; net PA6 and PA6/ EG compounds.

	net PA6	5 wt.%	10 wt.%	15 wt.%	20 wt.%	25 wt.%
dry	HB	HB	HB	HB	V2	V0
	t_1_: 5 ± 5 s	t_1_: 28 ± 4 s	t_1_: 17 ± 1 s	t_1_: 1 ± 0 s	t_1_: 0 ± 0 s	t_1_: 0 ± 0 s
	t_2_: 50 ± 7 s	t_2_: −5 ± 5 s	t_2_: 3 ± 1	t_2_: 9 ± 4 s	t_2_: 3 ± 1 s	t_2_: 0 ± 0 s
	C_ign_*: yes	C_ign_*: yes	C_ign_ *: yes	C_ign_*: yes	C_ign_*: yes	C_ign_*: no
humid cond.	V0	HB	HB	V2	V0	V0
	t_1_: 1 ± 0 s	t_1_: 47 ± 7	t_1_: 7 ± 7 s	t_1_: 7 s	t_1_: 0 ± 0 s	t_1_: 0 ± 0 s
	t_2_: −2 ± 0 s	t_2_: -	t_2_: 4 ± 2	t_2_: 13 s	t_2_: 4 ± 1 s	t_2_: 0 ± 0 s
	C_ign_ *: no	C_ign_ *: yes	C_ign_ *: yes	C_ign_ *: yes	C_ign_ *: no	C_ign_ *: no

* C_ign_: Cotton ignition.

**Table 3:** Cone calorimeter measurement—summary of main key figures.

ID.	PA6/EG(wt.%)	Tign (s)			pHRR (kW/m^2^)			THR (MJ/m^2^)		
**dry**		35	50	65	35	50	65	35	50	65
	100/0	273 ± 5	160 ± 3	122 ± 1	716 ± 34	706 ± 28	899 ± 43	114 ± 8	125 ± 1	119 ± 4
	95/5	245 ± 15	129 ± 4	103 ± 2	231 ± 15	386 ± 16	500 ± 41	85 ± 4	100 ± 7	108 ± 3
	90/10	233 ± 11	131 ± 1	109 ± 1	316 ± 12	359 ± 14	480 ± 39	94 ± 2	103 ± 2	113 ± 2
	85/15	194 ± 9	132 ± 3	107 ± 2	151 ± 20	167 ± 12	211 ± 22	14 ± 2	26 ± 2	40 ± 4
	80/20	185 ± 4	137 ± 1	111 ± 1	113 ± 12	122 ± 3	152 ± 10	10 ± 2	24 ± 2	30 ± 4
	75/25	188 ± 6	142 ± 2	112 ± 3	88 ± 7	70 ± 7	105 ± 7	7 ± 1	8 ± 2	23 ± 3
**cond.**										
	100/0	196 ± 1	133 ± 2	109 ± 2	351 ± 21	564 ± 34	871 ± 12	91 ± 7	92 ± 6	113 ± 3
	95/5	160 ± 6	119 ± 3	99 ± 1	276 ± 11	353 ± 5	453 ± 15	79 ± 8	88 ± 1	97 ± 2
	90/10	187 ± 0	122 ± 5	105 ± 2	278 ± 12	373 ± 11	462 ± 19	88 ± 1	98 ± 2	106 ± 1
	85/15	174 ± 6	128 ± 4	101 ± 0	124 ± 5	133 ± 5	183 ± 7	14 ± 2	23 ± 2	37 ± 4
	80/20	166 ± 1	129 ± 2	108 ± 4	58 ± 4	88 ± 5	136 ± 10	5 ± 0	16 ± 1	22 ± 1
	75/25	168 ± 5	133 ± 4	104 ± 1	49 ± 8	62 ± 10	140 ± 9	4 ± 1	8 ± 2	16 ± 3

## References

[1] Morgan A.B. (2019). The Future of Flame Retardant Polymers—Unmet Needs and Likely New Approaches. Polym. Rev..

[2] Schartel B. (2010). Phosphorus-based Flame Retardancy Mechanisms-Old Hat or a Starting Point for Future Development?. Materials.

[3] Focke W.W., Badenhorst H., Mhike W., Kruger H.J., Lombaard D. (2014). Characterization of commercial expandable graphite fire retardants. Thermochim. Acta.

[4] Huang J., Tang Q., Liao W., Wang G., Wei W., Li C. (2017). Green Preparation of Expandable Graphite and Its Application in Flame-Resistance Polymer Elastomer. Ind. Eng. Chem. Res..

[5] Weil E.D., Levchik S.V. (2008). Flame Retardants in Commercial Use or Development for Polyolefins. J. Fire Sci..

[6] Chen X., Wu H., Luo Z., Yang B., Guo S., Yu J. (2007). Synergistic effects of expandable graphite with magnesium hydroxide on the flame retardancy and thermal properties of polypropylene. Polym Eng. Sci.

[7] Dittrich B., Wartig K.-A., Hofmann D., Mülhaupt R., Schartel B. (2013). Carbon black, multiwall carbon nanotubes, expanded graphite and functionalized graphene flame retarded polypropylene nanocomposites. Polym. Adv. Technol..

[8] Dittrich B., Wartig K.-A., Hofmann D., Mülhaupt R., Schartel B. (2015). The influence of layered, spherical, and tubular carbon nanomaterials’ concentration on the flame retardancy of polypropylene. Polym. Compos..

[9] Mattausch H., Laske S., Hohenwarter D., Holzer C. The effect of mineral fillers on the rheological, mechanical and thermal properties of halogen-free flame-retardant polypropylene/expandable graphite compounds. Proceedings of the AIP Conference Proceedings.

[10] Schartel B., Braun U., Schwarz U., Reinemann S. (2003). Fire retardancy of polypropylene/flax blends. Polymer.

[11] Xu Y., Chen M., Ning X., Chen X., Sun Z., Ma Y., Qin J., Yu J., Zhang Z., Le Yang Bo X. (2014). The Thermal Stability and Flammability of Expandable Graphite-Filled Polypropylene/Thermoplastic Polyurethane Blends. J. Macromol. Sci. Part B.

[12] Zheng Z., Liu Y., Zhang L., Wang H. (2016). Synergistic effect of expandable graphite and intumescent flame retardants on the flame retardancy and thermal stability of polypropylene. J. Mater. Sci..

[13] Focke W.W., Kruger H.J., Mhike W., Taute A., Roberson A., Ofosu O. (2014). Polyethylene flame retarded with expandable graphite and a novel intumescent additive. J. Appl. Polym. Sci..

[14] Sun Z., Ma Y., Xu Y., Chen X., Chen M., Yu J., Hu S., Zhang Z. (2014). Effect of the particle size of expandable graphite on the thermal stability, flammability, and mechanical properties of high-density polyethylene/ethylene vinyl-acetate/expandable graphite composites. Polym. Eng. Sci..

[15] Tang M., Qi F., Chen M., Sun Z., Xu Y., Chen X., Zhang Z., Shen R. (2016). Synergistic effects of ammonium polyphosphate and red phosphorus with expandable graphite on flammability and thermal properties of HDPE/EVA blends. Polym. Adv. Technol..

[16] Wang H., Cao J., Luo F., Cao C., Qian Q., Huang B., Xiao L., Chen Q. (2019). Hugely enhanced flame retardancy and smoke suppression properties of UHMWPE composites with silicone-coated expandable graphite. Polym. Adv. Technol..

[17] Ge L.-L., Duan H.-J., Zhang X.-G., Chen C., Tang J.-H., Li Z.-M. (2012). Synergistic effect of ammonium polyphosphate and expandable graphite on flame-retardant properties of acrylonitrile-butadiene-styrene. J. Appl. Polym. Sci..

[18] Li Z., Qu B. (2003). Flammability characterization and synergistic effects of expandable graphite with magnesium hydroxide in halogen-free flame-retardant EVA blends. Polym. Degrad. Stab..

[19] Pang X.-Y., Tian Y., Shi X.-Z. (2017). Synergism between hydrotalcite and silicate-modified expandable graphite on ethylene vinyl acetate copolymer combustion behavior. J. Appl. Polym. Sci..

[20] Sover A., Marzynkevitsch S., Munack B. (2018). Processing Conditions of Expandable Graphite in PP andPA Matrix and their Performance. Mater. Plast..

[21] Alongi J., Frache A., Gioffredi E. (2011). Fire-retardant poly(ethylene terephthalate) by combination of expandable graphite and layered clays for plastics and textiles. Fire Mater..

[22] Wang G., Bai S. (2017). Synergistic effect of expandable graphite and melamine phosphate on flame-retardant polystyrene. J. Appl. Polym. Sci..

[23] Focke W.W., Muiambo H., Mhike W., Kruger H.J., Ofosu O. (2014). Flexible PVC flame retarded with expandable graphite. Polym. Degrad. Stab..

[24] Chung DD L. (2002). Review Graphite. J. Mater. Sci.

[25] Singh V., Joung D., Zhai L., Das S., Khondaker S.I., Seal S. (2011). Graphene based materials: Past, present and future. Prog. Mater. Sci..

[26] Yang X., Yang W., Fan J., Wu J., Zhang K. (2019). Effects of molding on property of thermally conductive and electrically insulating polyamide 6–based composite. J. Thermoplast. Compos. Mater..

[27] Babrauskas V., Peacock R.D. (1992). Heat release rate: The single most important variable in fire hazard. Fire Saf. J..

[28] Babrauskas V., Hurley M.J., Gottuk D.T., Hall J.R., Harada K., Kuligowski E.D., Puchovsky M., Torero J.L., Watts J.M., Wieczorek C.J. (2016). The Cone Calorimeter. SFPE Handbook of Fire Protection Engineering.

[29] Babrauskas V., Hurley M.J., Gottuk D.T., Hall J.R., Harada K., Kuligowski E.D., Puchovsky M., Torero J.L., Watts J.M., Wieczorek C.J. (2016). Heat Release Rate. SFPE Handbook of Fire Protection Engineering.

[30] Herrera M., Matuschek G., Kettrup A. (2000). Comparative studies of polymers using TA–MS, macro TA–MS and TA–FTIR. Thermochim. Acta.

[31] Levchik S.V., Weil E.D., Lewin M. (1999). Thermal decomposition of aliphatic nylons. Polym. Int..

[32] Herrera M., Matuschek G., Kettrup A. (2001). Main products and kinetics of the thermal degradation of polyamides. Chemosphere.

[33] Hornsby P.R., Wang J., Rothon R., Jackson G., Wilkinson G., Cossick K. (1996). Thermal decomposition behaviour of polyamide fire-retardant compositions containing magnesium hydroxide filler. Polym. Degrad. Stab..

[34] Dresselhaus M.S., Dresselhaus G. (1981). Intercalation compounds of graphite. Adv. Phys..

[35] Luo W., Li Y., Zou H., Liang M. (2014). Study of different-sized sulfur-free expandable graphite on morphology and properties of water-blown semi-rigid polyurethane foams. RSC Adv..

[36] Schartel B., Wilkie C.A., Camino G. (2017). Recommendations on the scientific approach to polymer flame retardancy: Part 2—Concepts. J. Fire Sci..

[37] Afanasov I.M., Savchenko D.V., Ionov S.G., Rusakov D.A., Seleznev A.N., Avdeev V.V. (2009). Thermal conductivity and mechanical properties of expanded graphite. Inorg Mater..

[38] Jia Y., He H., Yu P., Chen J., Tian S. (2018). Preparation and characterization of synergistically improved thermally conductive polyamide 6 with low melting point metal and low-temperature expandable graphite. Polym. Compos..

[39] Fang K., Chen Y.F., Zhang S.C., Sun H.R., Wang G.H., Sun X.K. (2016). The Effect of Particle Size of Expandable Graphite on the Properties of an Expandable Thermal Insulation Material. KEM.

[40] Liu J., Pang X., Shi X., Xu J. (2020). Expandable Graphite in Polyethylene: The Effect of Modification, Particle Size and the Synergistic Effect with Ammonium Polyphosphate on Flame Retardancy, Thermal Stability and Mechanical Properties. Combust. Sci. Technol..

[41] Salmeia K., Fage J., Liang S., Gaan S. (2015). An Overview of Mode of Action and Analytical Methods for Evaluation of Gas Phase Activities of Flame Retardants. Polymers.

[42] Naoshi S., Chihong L. Suppression Effect of Water Vapor on Flammability Limits of Hydrocarbon Fuels—A study on fire suppression by water mist. Proceedings of the 6th Asia-Oceania Symposium on Fire Science and Technology.

